# Molecular Markers and Targets in Melanoma

**DOI:** 10.3390/cells10092320

**Published:** 2021-09-05

**Authors:** Cristina Teixido, Paola Castillo, Clara Martinez-Vila, Ana Arance, Llucia Alos

**Affiliations:** 1 Department of Pathology, Hospital Clínic of Barcelona, University of Barcelona, Villarroel 170, 08036 Barcelona, Spain; PCASTILL@clinic.cat (P.C.); lalos@clinic.cat (L.A.); 2 August Pi i Sunyer Biomedical Research Institute (IDIBAPS), Rosselló 149, 08036 Barcelona, Spain; amarance@clinic.cat; 3 Department of Medical Oncology, Hospital Clínic of Barcelona, University of Barcelona, Villarroel 170, 08036 Barcelona, Spain; CMARTINEZV@clinic.cat; 4 Department of Medical Oncology, Althaia Xarxa Assistencial Universitària de Manresa, Dr. Joan Soler, 1–3, 08243 Manresa, Spain

**Keywords:** melanoma, molecular pathways, markers, target therapy, *BRAF*, *NRAS*, *KIT*, *NTRK*

## Abstract

Melanoma develops as a result of several genetic alterations, with UV radiation often acting as a mutagenic risk factor. Deep knowledge of the molecular signaling pathways of different types of melanoma allows better characterization and provides tools for the development of therapies based on the intervention of signals promoted by these cascades. The latest World Health Organization classification acknowledged the specific genetic drivers leading to melanoma and classifies melanocytic lesions into nine distinct categories according to the associate cumulative sun damage (CSD), which correlates with the molecular alterations of tumors. The largest groups are melanomas associated with low-CSD or superficial spreading melanomas, characterized by frequent presentation of the *BRAF*V600 mutation. High-CSD melanomas include lentigo maligna type and desmoplastic melanomas, which often have a high mutation burden and can harbor *NRAS*, *BRAF*non-V600E, or *NF1* mutations. Non-CSD-associated melanomas encompass acral and mucosal melanomas that usually do not show *BRAF*, *NRAS*, or *NF1* mutations (triple wild-type), but in a subset may have *KIT* or *SF3B1* mutations. To improve survival, these driver alterations can be treated with targeted therapy achieving significant antitumor activity. In recent years, relevant improvement in the prognosis and survival of patients with melanoma has been achieved, since the introduction of *BRAF*/*MEK* tyrosine kinase inhibitors and immune checkpoint inhibitors. In this review, we describe the current knowledge of molecular pathways and discuss current and potential therapeutic targets in melanoma, focusing on their clinical relevance of development.

## 1. Introduction

### 1.1. Epidemiology

Melanoma is the most aggressive and deadly skin cancer. Its incidence has increased steadily in the last decades, especially in the Caucasian population, posing a heightened challenge to the global healthcare system [1,2]. Relevant geographical variations exist, depending on the clinical phenotype, the genetic background of individuals, and the extent of ultraviolet (UV) radiation exposure [3]. Currently, it is one of the most frequent cancers in fair-skinned people, especially those with blond or red hair, who have light-colored eyes. Unlike other solid tumors, melanoma mainly affects young and middle-aged people [4]. Melanoma-related mortality has increased in parallel with the increase in the incidence rate over the years, reaching a mortality rate of one in four deaths [5]. Nevertheless, the therapeutic landscape of unresectable stage III and IV melanoma has been revolutionized by immunotherapies and targeted therapies. Both strategies have shown markedly improved survival compared with the use of chemotherapy (ChT) regimens [6]. Melanoma mortality has decreased significantly since the US Food and Drug Administration (FDA) approved ipilimumab in 2011, the first immune checkpoint inhibitor (ICI) to improve survival in the advanced setting [7,8], and vemurafenib, a v-raf murine sarcoma viral oncogene homolog B1 (*BRAF*) tyrosine kinase inhibitor, first in class [9,10].

### 1.2. Risk Factors

Melanoma develops from cutaneous melanocytes, located in the basal layer of the epidermis. UV radiation represents a major contributor to cutaneous melanomagenesis through its harmful effects on the skin and direct DNA damage [11], and it triggers the acceleration of tumorigenesis. Intense and intermittent sun exposure, as well as exposure to UV-A rays from artificial sources, has also been linked to an increased risk of melanoma development [12].

Host risk factors, such as the number of nevi, both congenital or acquired, genetic susceptibility, and a family history of melanoma, are relevant risk factors for the development of melanoma. About 25% of cutaneous melanomas arise from a nevus [13]. Polymorphisms of the melanocortin 1 receptor (*MC1R*) gene represent the most relevant gene for susceptibility to melanoma [14].

A family history of melanoma is present in 5–15% of patients with cutaneous melanoma, but true hereditary melanoma due to a transmitted genetic mutation is less common, such as familial atypical multiple mole-melanoma (*FAMMM*) syndrome and its variant, melanoma-astrocytoma syndrome. Germline mutations in cyclin-dependent kinase inhibitor 2A (*CDKN2A*) and, less common, mutations in cyclin-dependent kinase 4 (*CDK4*) are the most frequent genetic abnormalities identified in these families [15]. Other inherited conditions, such as xeroderma pigmentosum, familial retinoblastoma, Lynch syndrome type II, and Li–Fraumeni cancer syndrome, may also be related to an increased risk of melanoma development [16].

## 2. Molecular Pathways of Melanoma Development

Cancer results from uncontrolled cellular growth of malignant tumor cells caused by a combination of genetic alterations that lead to neoplastic transformation and escape from the inhibitory signals. Several steps in this process are known as the hallmark of cancers [17].

Several key molecular pathways have been discovered to be involved in the onset, proliferation, survival, progression, and invasion. In this section, we summarize the major signaling pathways that are currently known to be dysregulated and involved in melanoma disease.

### 2.1. MAPK Pathway

**Figure 1:**
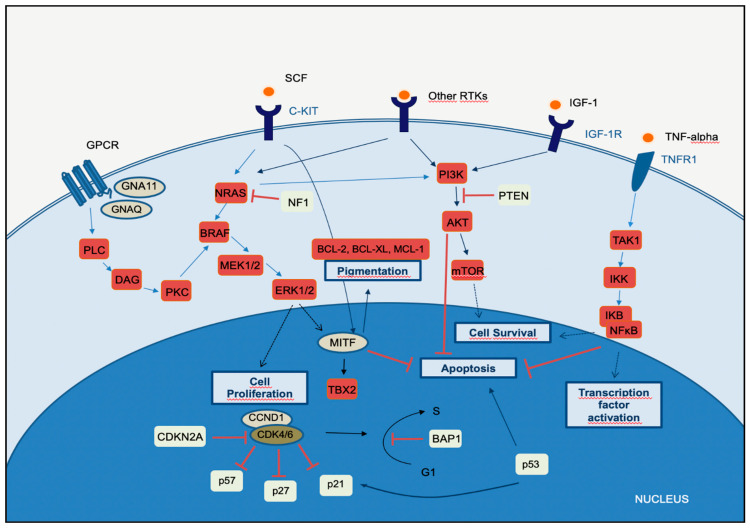
Melanoma key signaling pathways.

Melanomagenesis occurs after mutational events that produce signaling pathways critical for cell survival. Mitogen-activated protein kinase (*MAPK*) is a signal transduction pathway, involved in a variety of physiological programs, such as cell proliferation, differentiation, development, migration, apoptosis, and transformation, and is the most relevant in the development of melanoma (Figure 1) [18]. The *MAPK* pathway is activated by the binding of a growth factor to a receptor tyrosine kinase (RTK) on the cell surface and stimulates the guanosine triphosphatases (GTPase) activity of *RAS*. The signal propagates through the RAF, mitogen-activated protein kinase kinase 1 (*MAP2K1*), and extracellular signal-related kinase (*ERK*) cascade, which enters the nucleus to activate transcription factors and promote the cell cycle (Figure 1) [18].

The *MAPK*, *PI3K*, and *NFκB* pathways intersect significantly in melanoma pathogenesis. Briefly, in the *MAPK-ERK* pathway, stimulation of *GPCR* results in activation of PLC. This promotes DAG and then activates *PKC*, which stimulates the *MAPK* pathway. Receptor tyrosin kinases (RTKs) are activated by binding of extracellular growth factor ligands and activate the tyrosine kinase activity of the cytoplasmic domain receptor, starting the cascade of signals. Activated *RAS* activates the protein kinase activity of *RAF* isoforms (*RAF1, BRAF, ARAF*). Each *RAF* isoform possesses a distinct capacity to activate *MEK*, with *BRAF* being the strongest activator. *MEK* phosphorylates and activates downstream proteins, such as *ERK1* and *ERK2*. *ERK* can translocate to the nucleus and phosphorylate different transcription factors, which leads to the control of cell cycle progression. *MITF* is a target of *ERK* and controls the production of the pigment melanin, cell cycling, and survival. The binding of the ligand to *KIT* (SCF) results in activation of the *MAPK* and *PI3K* pathways. In the *PI3K-AKT* pathway, ligand binding to the RTK leads to dimerization and autophosphorylation of the receptor and activation. Activated RTK recruits *PI3K* to the plasma membrane. *PI3K* activates *AKT*, whereas *PTEN* antagonizes this process. *PI3K* may also be activated by *GPCR*, *IGF-1R*, and *RAS*. Both *ERK* and *AKT* activate the *mTOR*-signaling pathway, which mediates cell survival and proliferation. In the *TNFR* pathway (canonical *NF-κB* pathway), binding of the TNF-alpha cytokine to its receptor *TNFR1* results in *TAK1* activation. *TAK1* leads to the aggregation of a downstream kinase complex, the *IKK* complex. Phosphorylation of *IκB* by the *IKK* complex results in the release of *NFκB*. *NFκB* translocates to the nucleus and activates genes involved in cell survival and anti-apoptosis.

Fourteen *MAPKs* have been identified in mammals, and these kinases are typically divided into three main subfamilies: *ERKs*, c-Jun N-terminal kinases (*JNKs*), and *P38* kinases. Each of these *MAPKs* is activated through phosphorylation by an *MAPK* kinase (*MAP2K*), which in turn is activated by an *MAPKK* kinase (*MAP3K*) [18]. The *ERK* pathway is the best-characterized *MAPK* pathway, which has a relevant role in the development and progression of melanoma. On this *MAPK* axis, the role of *MAP3K* is played by the *RAF* family of serine/threonine kinases, which is characterized by an RAS/GTP-binding domain. RAS proteins vHa-ras Harvey rat sarcoma viral oncogene homolog (*HRAS*), *NRAS*, and v-Ki-ras2 Kirsten rat sarcoma viral oncogene homolog (*KRAS*) are small GTPases located in the plasma membrane that act as activators in several pathways, apart from *MAPK*. Additionally, activation signals via *RAS* on the inner surface of the cell membrane increase *ERK* activity. Consequently, there is an increase in cellular proliferation, greater cell survival, and resistance to apoptosis. Activated *ERK* can also induce the metastatic potential of melanoma through the expression of integrins that promote tumor invasion [19].

In melanoma, dysregulated *MAPK* signaling and sustained *ERK* activation can eventually lead to cascade hyperactivity and subsequent cell proliferation, survival, invasion, metastasis, and angiogenesis. The *BRAF* gene is frequently mutated in several cancers, and *BRAF*V600 is the most common mutation of the skin. Mutated *BRAF*V600 leads to elevated *BRAF* kinase activity and sustained activation of downstream targets, in addition to unresponsive negative feedback mechanisms [20]. The mutant *KRAS*Q61, the most frequent mutation of *KRAS* in melanoma, leads to an important decrease in its intrinsic hydrolytic activity and a sustained active state of *KRAS.* Mutations in other molecules may also lead to *RAS* overstimulation, such as loss-of-function mutations in neurofibromin 1 (*NF1*). In most melanomas with altered *NF1*, a loss-of-function mutation is found, in which neurofibromin loses its ability to inactivate *RAS* and promotes stimulation of the *RAF* and its downstream targets, leading to stimulation of the *MAPK* pathway and consequent cell proliferation and survival [21].

Telomerase reverse transcriptase (*TERT*) promoter mutations frequently occur in melanoma and, according to The Cancer Genome Atlas (TCGA) data, mainly in the mutated subtypes *BRAF* (75% of cases), *RAS* (72% of cases), and *NF1* (83% of cases), suggesting a link between *MAPK* activation and *TERT* expression. The active *MAPK* pathway promotes phosphorylation and activation of the ETS1 transcription factor by *ERK* (the mutated *TERT* promoter bears ETS-binding sites) [22].

### 2.2. PI3K-AKT Pathway

The phosphatidylinositol-3-kinases (*PI3Ks*) comprise a family of lipid kinases with regulatory roles in many cellular mechanisms, including cell survival and growth, differentiation, proliferation, transcription, and translation. The pathway transduces signals from a variety of growth factors and cytokines and is the major downstream effector of RTKs and G-protein-coupled receptors (*GPCRs*) (Figure 1). Activated *PI3K* leads to the formation of phosphatidylinositol-3,4,5-trisphosphate (PIP3) through phosphorylation of phosphatidylinositol-4,5-diphosphate (PIP2) in the plasma membrane. PIP3 is essential for the recruitment of the serine-threonine protein kinase *AKT* to the plasma membrane. *AKT* is crucial in this signaling pathway, transmitting signals by phosphorylating different downstream effector targets [23]. Once *AKT* is phosphorylated and fully activated, it turns on a major downstream effector of the *PI3K* pathway, inhibiting or activating a variety of targets and regulating important cellular processes, such as apoptosis, DNA repair, cell cycle, glucose metabolism, cell growth, motility, invasion, and angiogenesis. The main target of *AKT* is the mammalian target of rapamycin (*mTOR*), which has a central role in the *PI3K-AKT* pathway and cancer disease. *mTOR* plays a crucial part in regulating cell growth and proliferation by monitoring nutrient availability, cellular energy, oxygen levels, and mitogenic signals.

*PI3K-AKT* signaling has negative regulators, to control any persistent and long-term activation. A major regulator of *PI3K-AKT* signaling is the tumor suppressor phosphatase and tensin homolog (*PTEN*), which antagonizes the *PI3K* activity through its intrinsic lipid phosphatase activity, converting PIP3 back to PIP2. Loss of *PTEN* results in constitutive activation of *AKT* and has been largely associated with tumor development in malignant melanoma. Indeed, *PTEN* loss has been shown to be predictive of shorter overall survival (OS) [24,25].

The *PI3K* signaling cascade is upregulated in different types of cancer, including melanoma. More than two-thirds of primary and metastatic melanomas show high levels of phosphorylated *AKT*, suggesting that this alteration is an early event in melanoma pathogenesis. Oncogenic events that activate *PI3K-AKT* may include mutations or copy number variations in certain components of the pathway. *RAS* gene mutations and mutated or amplified expression of RTK may also hyperactivate the *PI3K-AKT* pathway [20]. Mutations in the *mTOR* gene are present in approximately 10% of melanomas, and this molecular event leads to shorter survival and worse prognosis [26]. *PI3K-AKT* signaling may also be activated in melanoma due to loss of function of the negative regulator *PTEN*, which occurs in 10–30% of cutaneous melanomas, leading to constitutive activation of the *PI3K* pathway. Interestingly, *PTEN* gene alterations are mutually exclusive with *NRAS* mutations, and approximately 20% of melanomas with loss of *PTEN* function also have *BRAF*V600E mutations [27].

### 2.3. CDKN2A, Cell Cycle, and Apoptosis Regulation

The *CDKN2A* gene encodes two proteins, p16^CDKN2A^ and p14^CDKN2A^, which have a tumor suppressor function. The cyclin proteins bind and activate CDKs, which has catalytic kinase activity. Several cyclin/CDK complexes have been identified that functionally act in different cell cycle phases: in the pre-replicative stage (G1), DNA duplication (S), and promotion of progression through the S phase to mitosis (Figure 1) [28]. p16^CDKN2A^ and p14^CDKN2A^ proteins have an inhibitory function, interfering with the activity of the cyclin/CDK complexes. p16^CDKN2A^ inhibits the cyclin D1 (*CCND1*)/*CDK4* complex, which, in turn, phosphorylates pRb and allows progression through the G1–S checkpoint. p14^CDKN2A^ is an antagonist of the mouse double minute 2 homolog (MDM2) protein. This protein degrades p53 and eliminates p53 control of cell growth. The p14^CDKN2A^ protein inhibits the oncogenic actions of *MDM2* by blocking its actions on p53 [28]. p53 is a transcription factor that functions as a major negative regulator of cell proliferation and survival. Inactivation of the *TP53* gene results in intracellular accumulation of genetic damage, which promotes melanoma development and progression. *TP53* can be inactivated through silencing or mutation, the latter occurring most frequently in high-cumulative solar damage-associated (CSD-associated) melanomas [29].

Somatic impairment of the *CDKN2A* gene in melanoma can occur by genetic deletions, inactivated mutations, or promoter hypermethylation and leads to a decrease of the function of p16^CDKN2A^ and/or p14^CDKN2A^ proteins, with consequent loss of cell cycle control. This situation is associated with a higher melanoma invasion potential and metastases [30].

As mentioned above, mutation of the *CDKN2A* gene at the germline level is the most frequent genetic alteration in patients with a strong familial history of melanoma. In addition, variants of the *MC1R* gene increase the melanoma risk in *CDKN2A* mutation carriers [31].

The *CCND1* and *CDK4* genes are found to be altered in a minority of melanomas, representing less than 5%, and depend on the melanoma type. *CCND1* gene amplifications affect about 30% of acral melanomas, 11% of lentigo maligna melanomas, and 6% of superficial spreading melanomas. *CDK4* gene amplification is frequently found in acral and mucosal melanomas [32].

### 2.4. MITF Pathway

The microphthalmia-associated transcription factor (*MITF*) acts as a master regulator of melanocyte development, function, and survival by modulating differentiation and cell cycle progression genes [33]. It is involved in the differentiation and maintenance of melanocytes and modulates melanocyte differentiation and pigmentation (Figure 1). In melanomas, *MITF* can behave as an oncogene, and in approximately 20% of melanomas, it amplifies and promotes the proliferation of tumor cells. Its amplification correlates with a worse prognosis and a lower OS and ChT resistance [33]. *MITF* is activated by the *MAPK* and cAMP pathways and regulates the transcription of three major pigmentation enzymes (TYR, TYRP1, and DCT) [34]. In melanoma, *ERK* activity stimulated by *BRAF* is associated with *MITF* ubiquitin-dependent degradation. *BRAF* can modulate intracellular MITF protein through two opposite mechanisms. On the one hand, it can degrade the MITF protein; on the other hand, *BRAF* can stimulate transcription factors that increase the expression of the MITF protein. About 10–15% of melanomas harbor the *BRAF* mutation along with *MITF* amplification, suggesting that additional mechanisms are involved in *ERK*-dependent degradation of *MITF*.

### 2.5. NFκB Pathway

The nuclear factor-kappaB (*NFκB*) is a pleiotropic transcription factor that regulates several genes involved in many critical pathways and, in addition to immune and inflammatory responses, participates in physiological conditions, development, and cancer initiation and progression [35]. There are five members of the *NFκB* family, which are distinguished by their Rel homology domain, the portion of the protein that controls DNA binding, dimerization, and interactions with IκB proteins: RelA (p65), RelB, c-Rel, p100/p52, and p105/p50 [36]. Most IκB proteins bind and inactivate *NFκB*, through the retention of *NFκB* in the cytoplasm. Cytoplasmic *NFκB* complexes remain transcriptionally inactive until the cell is stimulated. Activated *NFκB* translocates to the nucleus, where it binds to target DNA loci and induces transcription of a variety of target genes involved in cell survival and anti-apoptosis. UV irradiation promotes the inflammatory response and cytokine production in skin cells, and many of these cytokines have *NFκB* as their downstream target/effector. Sustained activity of *NFκB* may lead to exacerbated expression of pro-inflammatory mediators, leading to tissue damage that may evolve into organ dysfunction and eventually cancer. The canonical (classical) *NFκB* pathway is mainly activated by tumor necrosis factor (TNF)-alpha, IL-1, and Toll-like receptors: *TNFR*, *IL-1R*, and *TLR*, respectively (Figure 1) [37]. *NFκB* activation may also occur due to deregulations in upstream *MAPK* and *PI3K-AKT* signaling pathways through different mechanisms. In melanoma cells, these alterations lead to an increase in proliferation and resistance to apoptosis [38].

### 2.6. WNT Pathway

The WNT proteins compose a family of 19 glycoproteins that act through a variety of receptors to stimulate distinct intracellular sub-pathways. These pathways are involved in development, cell growth, migration, and differentiation. *WNT* signaling can be the canonical or non-canonical type. The canonical type includes the intracellular transcriptional co-activator *β-catenin* as a central component [39]. This pathway can be activated by WNT proteins, such as WNT1/WNT3A, through binding to Frizzled receptors (*FRZD1-7*) and co-receptors, lipoprotein receptor (*LRP*) 5 (*LRP5*) and *LRP6*, that stimulate intracellular signaling to finally regulate *β-catenin* stability and transcription. Stimulation of canonical *WNT* signaling activates and prevents *β*-*catenin* from degradation by inhibiting glycogen synthase kinase 3β (*GSK3β*). Blocking the degradation of *β-catenin* leads to its stabilization in the cytosol, allowing its translocation in the nucleus and association with transcription factors, such as lymphoid-enhanced transcription factor (*LEF*) and T cell transcription factor (*TCF*) [39]. Due to the binding of *β-catenin* to LEF/TCF, these factors become transcriptional activators, and *β-catenin*-containing complexes control the expression of several *WNT* target genes, including *c-MYC* and *CCND1*. Regulation of *c-MYC*, a well-characterized proto-oncogene, through the canonical *WNT* pathway involves the control of cancer cell metabolism. *c-MYC* functions as a transcription factor by binding to several target genes, many of which are involved in cell cycle control, including *CDKs*, and *CDK* inhibitors. In addition, canonical *WNT* signaling can cooperate with *MAPK* signaling to regulate *MITF* expression and activity, which is associated with melanoma cell proliferation. *WNT* signaling can also be activated through various non-canonical pathways, independent of *β-catenin*, which are less characterized [40].

## 3. The Integration of Histology and Molecular Diagnostics of Melanoma

**Figure 2:**
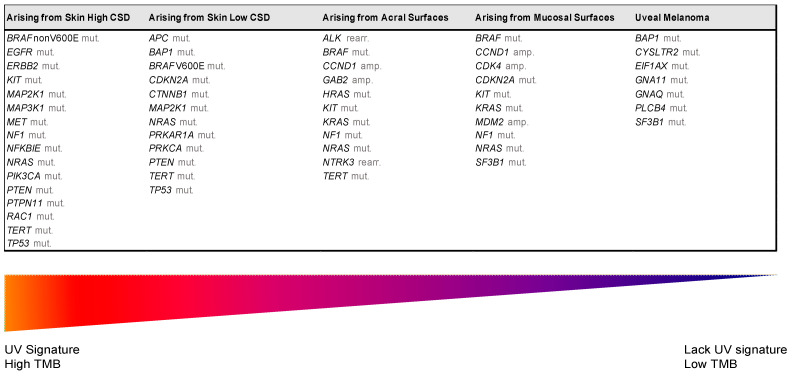
Genomic alterations of melanoma subtypes defined by UV exposure. Abbreviations: amp, amplification; CSD, cumulative sun damage; rearr, rearrangement; TMB, tumor mutational burden; UV, ultraviolet.

Despite recent molecular advances in melanoma characterization, paramount to diagnosis of a melanocytic skin lesion is the integration of several histopathological criteria with the clinical features. In many cases, general morphological criteria for atypia are often the subject of disagreement and inter-observer variability, especially in non-conventional lesions [41]. The World Health Organization (WHO) recognizes these challenges and incorporate the known molecular pathways in the latest WHO melanocytic tumor classification, introducing the concept of “intermediate” lesions. As stated in a recent review on the topic, this multidimensional classification showed that the view of melanocytic tumors as either benign or malignant might no longer be the proper approach [42]. Thus, WHO 2018 indicates nine categories/pathways leading to melanoma, each with specific genetic drivers (Table 1). Furthermore, melanomas can be clustered in three main subtypes, according to the degree of CSD (Table 1 and Figure 2) [43]. The largest group are melanomas associated with low-CSD or superficial spreading melanomas, which often arise on the trunk and proximal areas of the extremities. The most frequent molecular alteration in these melanomas is the *BRAF*V600E mutation [44]. In addition, *TERT* promoter mutations and *CDKN2A* mutations are also found in the majority of cases. *PTEN* and *TP53* are commonly observed in advanced tumors. Lentigo maligna and desmoplastic melanomas are considered tumors associated with high-CSD. These melanomas arise on heavily sun-damaged skin, such as the face or hands, and affect older people. Molecularly, they often have a high mutation load and may harbor *NRAS, BRAF* non-V600E, or *NF1* mutations. *TERT* promoter mutations and *CDKN2A* are also frequently found in these melanomas, and *KIT* mutations are found in a subset of cases. Interestingly, the number of mutations increases with the CSD grade (Figure 2), and desmoplastic melanomas harbor the highest tumor mutation burden. The category of “low to non-UV exposure/CSD” melanomas includes Spitz melanomas, acral melanomas, mucosal melanomas, melanomas developed from congenital nevi and blue nevi, and uveal melanomas. These melanomas rarely harbor *BRAF*, *NRAS*, or *NF1* mutations (triple wild-type) [43]. A subset of acral and mucosal melanomas may have *KIT* mutations, in addition to gene amplifications and structural rearrangements, most frequently of the *CCND1* gene and *SF3B1*. Therefore, genomic studies have subsequently exemplified that acral and mucosal melanomas are biologically distinct from their cutaneous counterparts at sun-exposed sites. Spitz melanomas show a particular oncogenic signaling pathway involving tyrosine kinase or serine-threonine kinase fusions, and melanomas in blue nevus and uveal melanomas are characterized by *GNA11* or *GNAQ* mutations [44].

Certainly, to reduce diagnostic uncertainties and maintain a diagnostic approach based on the WHO 2018 classification, histological assessment should be accompanied by basic immunohistochemistry (IHC) and molecular tests. Recent recommendations of the European Society of Pathology, the European Organization for Research and Treatment of Cancer, and the EURACAN for the diagnosis of intermediate melanocytic proliferations and melanoma variants indicate that most pathology laboratories should perform basic IHC tests, such as: HMB-45; SOX10; MITF, tyrosinase, MART-1; P16; Ki-67/MIB1; BAP1 (BRCA1-associated protein 1); β-catenin; PRAME; and at least one molecular method to detect *BRAF* codon 600 and *NRAS* mutations [42]. The most difficult cases that require complementary studies should be analyzed in specialized referral centers, where laboratories can determine a higher grade in a given lesion or the identification of molecular targets that can benefit from targeted therapy.

## 4. Therapeutic Targets and Current Treatment Strategies in Advanced Melanoma Patients

**Figure 3:**
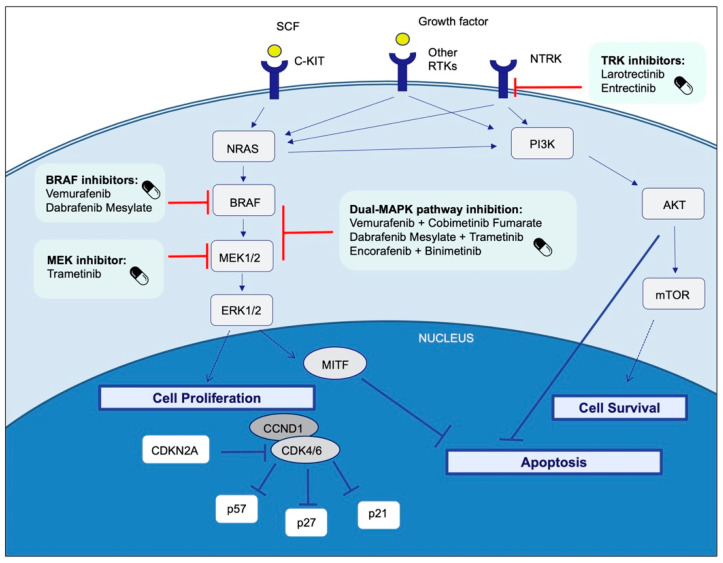
Melanoma US Food and Drug Administration-approved targeted therapy.

The progressive understanding of the molecular pathways of melanoma has enabled the development of successful immunotherapies and targeted therapies for unresectable stage III and IV melanoma. This section describes the most important targeted therapies for melanoma. Some of them have been approved for clinical use, or are in clinical trials or preclinical research (Figure 3 and Table 2 and Table 3).

### 4.1. BRAF

*BRAF* is the most important therapeutic target and the most frequent genetic alteration in cutaneous melanoma, affecting 40–60% of cases [20]. The most frequently found *BRAF* mutation is V600E, which affects about 80% of *BRAF*-mutated melanomas; V600K involves 15% of cases; and V600R/M/D/G mutations are found in about 5% of cases [45]. The *BRAF*V600E mutation is frequently associated with the superficial spreading subtype, younger patient age, and non-CSD skin sites, such as the trunk and proximal areas of the extremities. In contrast, V600K mutations correlate with CSD skin sites, such as the head and neck, and patients of older age [46].

The presence of *BRAF* mutations in nevi supports the hypothesis that activation of the *RAF/MEK/* pathway is an early event in melanoma development [47].

The FDA approved *BRAF*V600E/K inhibitors, *MEK*1/2 inhibitors, and dual-*MAPK* pathway inhibition with a combination of *BRAF* and *MEK* inhibitors for patients with *BRAF*V600E/K mutation-positive unresectable or metastatic melanoma. Furthermore, a combination of dabrafenib plus trametinib is approved for adjuvant treatment of resected stage III *BRAF*V600E/K mutant melanoma. On the other hand, larotrectinib and entrectinib are approved for patients with solid tumors that have *NTRK* gene fusions.

#### 4.1.1. *BRAF* and *MEK* Inhibitors

Selective *BRAF* inhibitors have demonstrated remarkable clinical activity in melanoma patients carrying *BRAF*V600 mutations [93], such as vemurafenib [9,57], first in class. Its FDA approval in this setting was based on the results of the BRIM3 phase III trial, in which patients treated with vemurafenib had an OS and progression-free survival (PFS) of 13.6 and 5.3 months, compared to dacarbazine-treated patients, who had 9.7 and 1.6 months, respectively [9] (Table 2). Additionally, another selective *BRAF* inhibitor (*BRAF*i), dabrafenib, showed efficacy in this group of patients in the BREAK3 phase III trial, in which patients treated with dabrafenib had an OS and PFS of 20 and 6.9 months, whereas those treated with standard chemotherapy had 15.6 and 2.7 months, respectively [53]. Based on these findings, the FDA also approved dabrafenib as a first-line treatment for unresectable or metastatic melanomas carrying the *BRAF*V600 mutation.

Importantly, *BRAF*i leads to characteristic side effects, including photosensitivity, which can limit treatment, and the rapid development of cutaneous squamous cell carcinoma (cuSCC) or other keratinocytic secondary neoplasias, which are thought to arise due to the paradoxical activation of the *MAPK* pathway in keratinocytes that are wild-type for *BRAF* but present upstream *RAS* activation in chronically damaged skin [94]. Nonetheless, patients can develop resistance through upregulation of RTKs or *NRAS* [95,96]. Preclinical data showed that *BRAF*i-resistant cells were sensitive to *MEK* inhibitors (*MEK*i) [97]. Thus, the combination of *BRAF* and *MEK* inhibitors (*BRAF*i plus *MEK*i) was predicted to decrease this side effect, and indeed, this combination treatment was not only demonstrably linked to improved PFS and OS compared to *BRAF*i monotherapy, but also decreased the development of cuSCC. Therefore, following the discovery of the clinical activity of single-agent *MEK*i, the use of combinations of *BRAF*i plus *MEK*i was evaluated in clinical studies. Currently, three combinations of *BRAF*i plus *MEK*i have been approved for clinical practice and appear to be comparable in terms of efficacy, but to date, no adequate direct comparison has been performed in randomized trials.

- Dabrafenib plus trametinib

The combination of *BRAF*i dabrafenib and *MEK*i trametinib was the first combination of *BRAF*i plus *MEK*i approved for the treatment of advanced melanoma in the United States in 2014 and in the European Union in 2015. The approval was based on the results of the COMBI-v trial [54,98], which compared dabrafenib plus trametinib to vemurafenib, and the COMBI-d trial [55,99], which compared dabrafenib plus trametinib to dabrafenib monotherapy (Table 2). In the phase III COMBI-d trial, the overall response rate (ORR) was 69% for the combination and 53% for dabrafenib alone [54]. In the phase III COMBI-v trial, ORR was 64% for the combination and 51% for vemurafenib alone. Rates of severe adverse events (AEs) and study drug discontinuation were similar in both arms. The 5-year OS rate for both pooled trials was 34% for the combination arm. Further, 19% of patients treated with *BRAF*i plus *MEK*i achieved a complete response, while for these patients, the OS rate was 71% at 5 years [100].

- Cobimetinib plus vemurafenib

Cobimetinib is another *MEK*i developed for the treatment of advanced melanoma in combination with *BRAF*i vemurafenib. In the phase III CoBRIM study, 495 patients with previously untreated advanced melanoma were randomized to receive either vemurafenib plus cobimetinib, or vemurafenib alone. ORR was 68% for vemurafenib plus cobimetinib and 45% for vemurafenib alone (Table 2). The median OS was 22.3 months for the combination versus 17.4 months for vemurafenib [56,101]. Extended follow-up demonstrated a 4-year OS rate of 35% in the combination group versus 29% in the control group [102]. The toxicity profile of vemurafenib plus cobimetinib differs from that of dabrafenib plus trametinib. Diarrhea, nausea, fatigue, rash, liver enzyme abnormalities, and photosensitivity (caused by vemurafenib) are more likely to occur with vemurafenib plus cobimetinib, while pyrexia is more likely to develop with dabrafenib plus trametinib [102].

- Encorafenib plus binimetinib

Nowadays, a third combination of a *BRAF*i plus *MEK*i, encorafenib and binimetinib, is also authorized based on the results of the three-arm phase III COLUMBUS trial. In this trial, 577 patients with advanced melanoma with the *BRAF*V600 mutation were randomized to encorafenib plus binimetinib, encorafenib, or vemurafenib monotherapy [57]. The results were best for the combination group, with an ORR of 63% versus 51% versus 40% and a median OS of 33.6 months versus 23.5 months versus 16.9 months (Table 2). The most common grade 3 or 4 treatment-related adverse events (TRAEs) reported for the combination were increased glutamyltransferase (9%), increased creatine phosphokinase (7%), and hypertension (6%).

#### 4.1.2. Differences between *BRAF*V600E and *BRAF*V600K Mutations

The most common *BRAF*V600 mutations are V600E (80%) and V600K (14%). Even though individual phase III targeted therapy trials have not performed a direct comparison between *BRAF*V600E and *BRAF*V600K-mutant melanomas, in three separate trials, *BRAF*V600K melanomas had a numerically lower response rate and shorter median PFS with *BRAF*i compared with V600E melanomas, and two pooled analyses of *BRAF*i plus *MEK*i showed shorter PFS in multivariate analysis [103,104].

#### 4.1.3. Resistance Mechanisms to *BRAF* and *MEK* Inhibition

Despite these promising results, almost all patients diagnosed with *BRAF*-mutated advanced melanoma will develop tumor relapse within several months after initiation of *BRAF*i +/− *MEK*i treatment. Different mechanisms of drug resistance underlie the progression of the disease and the activation of both the *MAPK* and *PI3K-AKT* pathways. Inhibition of *BRAF* leads to increased *RAS* activity, which in turn can activate the *PI3K-AKT* signaling pathway. Furthermore, it has been observed that patients with melanoma carrying *PTEN* mutation/loss, treated with dabrafenib, have a shorter median PFS [105]. Among the acquired resistance mechanisms, mutations in the *NRAS* and *MAP2K* genes determine the dependence of the *MAPK* pathway [106].

*NF-1* inhibits *RAS* activity under physiological conditions, so its role in resistance to *BRAF-*targeted therapy has been widely studied [107]. It was demonstrated that *NF-1* loss-of-function mutations can lead to continuous *RAS* activation, which can activate both *MAPK* and *PI3K-AKT* signaling pathways downstream and confer resistance to target therapy. The *BRAF* oncogene cooperates with *CDKN2A*, inducing its upregulation, and consequently, cell arrest and senescence. Thus, *CDKN2A* inactivation could induce melanoma progression in a *BRAF*-mutated nevus. Nathanson et al. demonstrated that a lower copy number of *CDKN2A* was significantly associated with decreased PFS in patients treated with dabrafenib, while a higher *CCND1* copy number was significantly associated with a worse prognosis and resistance to *BRAF*i [105].

Apart from these mechanisms that reactivate *RAS*, there are structural changes in oncogenic *BRAF* due to aberrant splicing that can lead to resistance. For example, p61-*BRAF*V600E splice variants retain active kinase activity but are unable to bind *RAS*. They dimerize regardless of *RAS* status and drive constitutive signaling to *ERK*, uncoupled from upstream regulation [108]. Importantly, tumor heterogeneity and heterogeneous mechanisms of resistance are present in tumors and at different metastatic sites simultaneously [109]. Recent efforts to dissect the degree of heterogeneity and its clinical implications have led to transcriptional studies targeting thousands of single tumor cells, and show that at the cellular level in all tumors, there are distinct transcriptional patterns within each tumor that display varying degrees of predicted responsiveness to *BRAF* inhibition [110].

Finally, ongoing studies are investigating the addition of *CDK4/6* inhibitors, *PI3K/mTOR*, and ICIs to combat resistance [96,111] (Table 3). Additionally, RhoA GTPases are emerging as a potential pathway for *BRAF* resistance, since preclinical data has shown that inhibition of the pathway by Rho kinase inhibitors promotes resensitization to *BRAF*-targeted therapy [112].

### 4.2. Immune Checkpoint Inhibitors: Anti-CTLA-4 and Anti-PD1

Treatment and prognosis of metastatic melanoma has changed radically in the last decade since the approval of ICIs, those directed to protein 4 associated with cytotoxic T lymphocytes (CTLA-4) and programmed cell death-1 (PD1). Both are inhibitory receptors that regulate immune responses by different mechanisms. CTLA-4 is a negative regulator of T cells and is expressed by naive T cells, which leads to a robust inhibitory signal during T cell activation when it binds to costimulatory protein B7 in antigen-presenting cells in the lymph node. On the other hand, anti-PD1 inhibitors prevent the binding of PD1 and its ligands (PD-L1 and PD-L2) to produce an effective immune response. When PD1 receptor binds to its ligands, it works to decrease the ability of already activated T cells to produce an effective immune response and prevent the immune system from rejecting the tumor [113].

#### 4.2.1. Anti-CTLA4

In patients with metastatic melanoma, phase III clinical trials of ipilimumab, a fully human IgG1 monoclonal antibody inhibiting CTLA-4, demonstrated a significant improvement in PFS and OS when compared with a gp100 vaccine [7] or dacarbazine ChT [48].

#### 4.2.2. Anti-PD1-Based Therapies

In 2014, the FDA approved pembrolizumab and nivolumab as the first anti-PD1 (CD279)-directed monoclonal antibodies for advanced or metastatic melanoma. Both drugs received EU approval in 2015. Approval of nivolumab was based on the results of the CheckMate 066 phase III study, in which *BRAF* wild-type advanced melanoma patients were treated with nivolumab or dacarbazine (DTIC) [49]. In this trial, the median OS was 37.2 months for nivolumab versus 11.2 months for dacarbazine, and the 1- and 2-year OS rates were 73% and 58%, respectively, for nivolumab-treated patients (Table 3). Three months prior to nivolumab, pembrolizumab was approved by the FDA for the treatment of metastatic melanoma. The accelerated approval was based on the results of an activity-estimating cohort conducted within the phase Ib KEYNOTE-001 trial [114]. Pembrolizumab showed 5-year OS rates of 34% in all patients and 41% in treatment-naïve patients with melanoma [115]. Furthermore, in a phase III trial of pembrolizumab versus ipilimumab, the 2-year OS rates were 55% versus 43%, respectively [52,114]. The efficacy of pembrolizumab and nivolumab has never been directly compared in patients with metastatic melanoma. In a retrospective study, OS from 888 patients with metastatic melanoma treated with first-line pembrolizumab or nivolumab was compared, with no statistical difference [116].

- Combination of Anti-PD1 with Anti-CTLA-4 Monoclonal Antibodies

The combination of CTLA-4 and PD1 blockade is supposed to synergistically stimulate the immune response against cancer cells. Approval of dual therapy was based on data from the phase III CheckMate 067 trial, in which combination therapy with anti-CTLA-4 and anti-PD1 blockade demonstrated superior clinical activity, with an ORR ranging from 50 to 60% and improved OS compared to ipilimumab, despite increased toxicity with the combination treatment [50]. In this phase III trial, a total of 945 previously untreated patients with unresectable stage III or IV melanoma were included. With a minimum follow-up of 60 months, median OS had not been reached in the combination group and was 36.9 months in the nivolumab group, as compared with 19.9 months in the ipilimumab group [117].

Moreover, an important trial to better elucidate the contribution of each combination therapy compound to toxicity is the CheckMate 511 trial, conducted by Lebbé et al. [51]. CheckMate 511 compared ‘standard’ regimen doses of 3 mg/kg ipilimumab and 1 mg/kg nivolumab as used, e.g., in CheckMate 067 with a 1 mg/kg ipilimumab and 3 mg/kg nivolumab regimen (Table 2). Results showed decreased toxicity but similar efficacy for the reduced ipilimumab and increased nivolumab regimen. It should be mentioned that the study was only powered with respect to the toxicity comparison but not efficacy. Therefore, the outcomes of this trial supported the assumption that the immunotoxicity of the combination can be reasonably moderated with a reduced dose of ipilimumab. In summary, dual therapy with ipilimumab and nivolumab appears to be superior to either anti-CTLA-4 monotherapy or anti-PD1 monotherapy [6].

- Novel Combinations of *BRAF*/*MEK* Inhibitors and Immune Checkpoint Inhibitors

Given the rapid and deep responses seen with *BRAF*i plus *MEK*i and the durable responses observed with ICIs, the combination of these therapeutic strategies appears promising. The phase III IMspire 150 trial evaluated the addition of atezolizumab, an anti-PD-L1 monoclonal antibody, to vemurafenib and cobimetinib in patients with *BRAF*V600-mutant metastatic melanoma [58]. In total, 514 patients were randomly assigned to receive atezolizumab, vemurafenib and cobimetinib or placebo, vemurafenib and cobimetinib, as first-line therapy (Table 2). The study met its primary endpoint, PFS. The triple therapy did not increase ORR. Grade 3 or 4 TRAEs occurred in 79% of patients treated with the triple combination and in 73% of patients treated with only vemurafenib plus cobimetinib, so it was concluded that the addition of atezolizumab to targeted therapy with vemurafenib and cobimetinib was tolerable and significantly increased PFS in patients with *BRAF*V600-mutant metastatic melanoma.

Another triple therapy that has been explored is the addition of a new monoclonal antibody anti-PD1, spartalizumab, to dabrafenib and trametinib, in the phase III COMBI-I trial, compared to dabrafenib and trametinib plus placebo, as first-line therapy in patients with *BRAF*V600E/K-mutant advanced melanoma [59]. With ORR for the triple therapy being slightly higher (69%) compared to the double (64%), the trial unexpectedly did not meet its primary endpoint of investigator-assessed PFS for patients treated with the triple therapy (Table 2).

Finally, on this topic, there is some preclinical evidence that in *BRAF* wild-type melanoma, the combination of *MEK*i with ICIs may enhance the antitumor effects. Preclinical data has shown that *MEK*i cobimetinib inhibits *MAPK* signaling and increases immune cell infiltration in the tumor, providing a strong rationale for combining cobimetinib with the anti-PD-L1 antibody atezolizumab [118]. Based on these findings, IMspire 170, a phase III trial, randomized 446 patients with advanced *BRAF* wild-type melanoma to receive either the *MEK*i cobimetinib plus atezolizumab, or the anti-PD1 monoclonal antibody pembrolizumab alone. The trial did not meet its primary endpoint, showing a median PFS of 5.5 months with cobimetinib plus atezolizumab versus 5.7 months with pembrolizumab alone.

- Biomarkers of response and resistance mechanisms to immune checkpoint inhibitors

Analysis of TCGA data from melanoma cases revealed that cutaneous melanoma displays a high mutational burden and UV signature (Figure 2) [119]. In addition to neoantigen recognition, a high mutational load was also found to correlate with clinical benefit to ICIs [120]. Similarly, a positive correlation was observed between a higher mutational load and increased CD8-positive T cell infiltration. Furthermore, PD-L1-positive patients treated with pembrolizumab had increased PFS, OS, and ORR [50]. Nevertheless, some patients with PD-L1-positive tumors do not respond to PD1 blockade, and conversely, some patients with PD-L1-negative tumors respond [121]. Together, the aforementioned data indicate that PD-L1 expression is a possible surrogate for lack of immunogenicity, as well as other failures further down the immune cycle, but is a suboptimal biomarker to predict response to ICIs in melanoma patients [122]. Cooperating with the loss of antigenicity and the intrinsic characteristics in the tumor that render them more or less vulnerable to immunotherapy are additional mechanisms arising during the immune response that directly inhibit tumor-targeting T cells. For example, resistances can be acquired by new resistance-driving mutations in genes involved in interferon-receptor signaling, antigen presentation, and the *β-catenin* signaling pathway [123,124,125]. Gene expression signatures associated with responses to immunotherapy are an area of active research. The *β-catenin* signaling pathway signature and the 10-gene interferon-gamma (*IFNG*) signature have been shown to predict resistance and responses to ICIs in melanoma [123]. Ayers et al. analyzed gene expression profiles (GEPs) using RNA from baseline tumor samples from patients treated with pembrolizumab. A 10-gene “preliminary IFNG” signature (*IFNG*, *STAT1*, *CCR5*, *CXCL9*, *CXCL10*, *CXCL11*, *IDO1*, *PRF1*, *GZMA*, and *MHCII HLA-DRA*) was constructed that was able to separate anti-PD1 (pembrolizumab) responders and non-responders, among the 19 pilot data patients with melanoma [126]. In the same study, data showed that such a signature might perform favorably compared with PD-L1 IHC in PD-L1-unselected populations.

### 4.3. Other Immunotherapy Treatment Strategies: Adoptive Cell Therapy

Patients who progress after anti-PD1 therapy, anti-PD1 plus anti-CTLA-4 therapy, and targeted agents have limited options. Only 4–10% of these patients have objective responses to ChT, with a limited median OS of 7 months [127,128]. New approaches to cancer immunotherapy have raised hope for effective treatments. Recently, adoptive cell therapy (ACT) has been recognized as a method to provide a long-lasting and effective response in melanoma. As its name implies, ACT is designed to redirect host lymphocyte cells against tumor cells [129]. We can distinguish three ACT strategies: tumor-infiltrating lymphocytes (TILs), T cell receptor-engineered T cells (TCR-T), and chimeric antigen receptor T cells (CAR-T).

#### 4.3.1. Tumor-Infiltrating Lymphocytes

TILs are autologous CD4 and CD8 T cells in the tumor microenvironment, which have the potential to recognize tumor-specific antigens [130]. However, due to chronic antigenic stimulation, they are converted to an “exhausted” state and become functionally impaired. In 1988, Rosenberg and colleagues developed a method to reactivate these cells. Promising response rates were achieved by isolating autologous TILs from a resected metastasis, in vitro expansion in the presence of interleukin (IL)-2, and reinfusion of cells followed by boluses of IL-2 [131]. A recent meta-analysis by Dafni and colleagues found an ORR of 41% and a complete response rate of 12% in a total of 410 patient treated with TILs [130]. Recently, phase II results evaluating the efficacy of lifileucel (an autologous TIL product) in patients with advanced melanoma who had been previously treated with ICIs and *BRAF/MEK*-targeted agents observed an ORR of 36% and in patients refractory to anti–PD1/PD-L1 an ORR of 41% [132]. Additionally, a randomized phase III trial (NCT02278887) that is currently recruiting aims to show a better survival rate after TIL infusion compared to standard-of-care ipilimumab in patients with advanced-stage progressive melanoma in the first line of anti-PD1 treatment.

#### 4.3.2. T Cell Receptor-Engineered T Cells

The limitations of TILS treatment have prompted the development and use of TCR-T and CAR-T. TCR-T are manufactured from autologous peripheral blood mononuclear cells collected by apheresis, after which CD3 cells are genetically modified and shortly expanded in vitro. In 2006, the first trial targeting the melanoma differentiation antigen MART-1 showed feasibility but only low clinical response rates (2/17) [133]. More encouraging results were seen in subsequent trials with MART-1 and gp100-reactive TCR-T, with ORR in 30% and 19% of patients, respectively [134]. Despite the encouraging results, the downside of targeting antigens shared between melanoma cells and melanocytes is the risk of inducing on-target, off-tumor toxicities [135]. So far, no severe toxicities have been reported after treatment with high-affinity NY-ESO-1-specific TCR gene-modified T cells, whereas encouraging response rates of up to 55% and an estimated 3–5-year OS rate of 33% have been achieved in a phase II trial [136].

#### 4.3.3. Chimeric Antigen Receptor T Cells

A CAR is a fully synthetic antigen receptor. CAR-T can be manufactured from T cells, which are collected by apheresis, genetically modified to express the CAR, and expanded over 7 to 10 days [137]. As a consequence, they can be deployed to treat patients regardless of their HLA-type and can circumvent mechanisms of tumor resistance, such as MHC downregulation and defective antigen processing [137]. A phase I/II study evaluating vascular endothelial growth factor receptor 2 CAR-T (NCT01218867) in patients with metastatic cancer, including melanoma, was discontinued due to a lack of objective responses [138]. Currently, there are three phase I dose escalation trials enrolling patients with melanoma (NCT03893019, NCT03635632, NCT04119024).

### 4.4. NRAS

Unlike *BRAF*, *NRAS* is not a therapeutic target. *NRAS* and *BRAF* mutations are usually mutually exclusive, but their coexistence has been described [139]. *NRAS* mutations are present in about 10–25% of cutaneous melanomas, with preference in the nodular melanoma type and melanomas arising in the CSD skin, suggesting that it may be a consequence of UV-related mutagenesis. Several studies suggested that *NRAS* mutations are significantly related with a worse prognosis in melanoma patients [140,141]. However, controversial data exists on the prognostic role of *NRAS* mutation in melanoma patients. In a phase III study [118], which evaluated the combination of treatment with atezolizumab and cobimetinib versus pembrolizumab monotherapy in *BRAF* wild-type advanced melanoma patients, no differences were found with respect to the mutational status of *NF1* or *NRAS* or no mutation as determined by a next-generation sequencing platform in terms of prognostic and treatment efficacy.

Effective treatment options in *NRAS*-mutated advanced melanoma are urgently needed, especially after failure of immunotherapy with anti-CTLA4 or anti-PD1 antibodies. Binimetinib, an *MEK* inhibitor, has shown clinical activity (20% partial response) in an open-label phase II study in this group of patients [142]. Based on these results, a phase III trial (NEMO) was conducted, including 402 patients harboring an *NRAS*Q61R, Q61K, or Q61L mutation who had not been previously treated or whose disease had progressed during or after immunotherapy, and randomized 2:1 to receive either binimetinib or dacarbazine [143]. PFS was significantly longer in the binimetinib group, but no differences were observed for OS. Other *MEK* inhibitors (trametinib and selumetinib) have been evaluated in *NRAS*-mutated advanced melanoma patients, without favorable outcomes [144,145]. In contrast, another *MEK* inhibitor in clinical development, pimasertib (Table 3), in a phase I study showed clinical activity in patients with locally advanced or metastatic melanoma, particularly in tumors with *BRAF* and *NRAS* mutations. The ORR was 12.4% (11/89), and 9 of the 11 responders presented a *BRAF* and/or *NRAS* mutation (3/11) [74].

### 4.5. C-KIT

*C-KIT* is an RTK directly responsible for binding to growth factors and initiating the *MAPK* and *PI3K-AKT* pathways (Figure 1). Mucosal melanoma and acral melanomas are the types of melanoma harboring the highest *C-KIT* alterations, followed by melanomas that arise on CSD skin. Indeed, *C*-*KIT* mutations and amplifications are detected in 10–15% of acral melanomas, 20–30% of mucosal melanomas, and about 1% of other cutaneous melanomas [32]. *C-KIT* is a therapeutic target in gastrointestinal stromal tumors with activating mutations of *C-KIT*, but in melanoma, it is under study, with no approved drugs yet. Advanced melanomas with *C-KIT* alterations have shown efficacy in terms of ORR with the *C-KIT* inhibitors imatinib (ORR 23.3%) and nilotinib (ORR 26.2%) in phase II trials [64,65,146] (Table 3).

### 4.6. GNAQ/GNA11

Cutaneous melanomas arising in blue nevus, as well as the uveal melanoma subtype can harbor *GNAQ* or *GNA11* mutations, and their identification helps improve the differential diagnosis of melanocytic lesions. *GNAQ* mutations have been detected in about 90% of blue nevus, 50% of malignant blue nevus, and 50% of primary uveal melanoma. *GNA11* mutations have been observed in less than 10% of blue nevus, one third of primary uveal melanomas, and about 60% of metastatic uveal melanomas [43,147].

Several studies have been conducted to evaluate the efficacy of specific agents in melanomas harboring *GNAQ/GNA11* mutations (Table 3). In vitro, tumors harboring *GNAQ/GNA11* mutations were found to respond to *MEK* inhibitors, but the combination of *MEK/PI3K* inhibitors has shown increased activity [148]. On the other hand, a phase Ib dose-escalation study of the *MEK* inhibitor with the dual *PI3K/mTOR* inhibitor has been proposed as a potential treatment in these tumors, but the combination was poorly tolerated, and responses were minimal.

### 4.7. SF3B1

*SF3B1* mutations have been identified in subsets of solid tumors, as well as in myelodysplastic syndrome and chronic lymphocytic leukemia. Recently, *SF3B1* was identified as a significantly mutated gene in uveal melanoma (20%) and mucosal melanoma (42%), especially in female genital and anorectal melanomas [149]. *SF3B1* encodes for a part of the spliceosome, splicing factor 3 subunit 1. *SF3B1* mutations are the most common spliceosomal component gene mutation implicated in the pathogenesis of cancer and act by causing aberrant RNA splicing events [150]. The clinical relevance of these splicing events is not completely clear, but *SF3B1*-mutated tumors have been shown to be at risk of metastasis. *SF3B1* mutations are being studied as a potential therapeutic target using *SF3B*-small-molecule inhibitors [151]. However, the phase I studies performed did not achieve a good response rate (Table 3) [91,92].

### 4.8. NTRK

Neurotrophic tyrosine receptor kinase (*NTRK*) comprises a family of three genes encoding tropomyosin receptor kinases (*TRK*): *TRKA*, *TRKB*, and *TRKC* receptors, which correspond to the *NTRK1*, *NTRK2*, and *NTRK3* genes, respectively. These receptors play an important role in the development and function of neuronal tissue [152]. Gene fusions represent the major molecular aberration involving *NTRK* in tumorigenesis and have been found in several cancers displaying different histologic phenotypes, including many different epithelial tumors, glioblastomas, and sarcomas. In melanoma, *NTRK* fusions are uncommon, usually found in Spitz melanomas (Table 1) [153]. Furthermore, *NTRK* translocations have also been identified in 2.5% of acral melanomas [154] and less than 1% of cutaneous and mucosal melanomas [155].

Targeting *NTRK* fusions represents a new opportunity for cancer treatment, as selective inhibitors (entrectinib and larotrectinib) have been developed and are FDA approved for adults and pediatric patients with solid tumors that harbor a *NTRK* gene rearrangement without a known acquired resistance mutation, that are either metastatic or unresectable, and who have no satisfactory alternative treatments or whose cancer has progressed following treatment (Figure 3).

The *NTRK* inhibitors entrectinib and larotrectinib have demonstrated a 57% and 79% ORR, respectively, in tumors with *NTRK* family fusions, regardless of histology [156,157]. Given this efficacy, these predictive biomarkers would be worth testing in tumors from patients diagnosed with metastatic melanomas that do not respond to other treatments.

### 4.9. Other Therapeutic Options

- NFκB pathway

Inhibition of *NFκB* can promote benefits by enhancing apoptosis, and this may be achieved by targeting crosstalk regulators of the *PI3K-AKT* and *MAPK* pathways [158].

Proteasome inhibitors, such as bortezomib, have been used to inhibit *NFκB* by preventing the degradation of IκB. These inhibitors have been shown to induce a decrease of melanoma proliferation in vitro [159,160]. Bortezomib also induces a reduction of tumor growth in vivo but has shown significant toxicity after clinical trials [161].

Recent efforts have been directed at the development of selective inhibitors of the *NFκB* pathway. BMS-345541, a specific *IKK* inhibitor, has been shown to reduce constitutive *IKK* activity and apoptosis of melanoma cells (Table 3) [83]. The NBD (NEMO-binding domain) peptide can bind to *NEMO* and prevent its interaction with *IKKα/β*, which is crucial for *IKK* complex activity and activation of *NFκB* (Figure 1). The use of NBD peptides can promote a significant decrease in proliferation and apoptosis in human melanoma cell lines, but neither NBD peptides nor BMS-345541 have reached clinical trials yet [162].

Highly specific inhibitors of *NFkB* may be used to minimize the pleiotropic effects of nonspecific inhibitors and to reduce toxicity in vivo. This field of research is new, with great potential, and could provide more alternative options for the treatment of melanoma in the future.

- *WNT* pathway

Several inhibitors of the *WNT* pathway of natural or synthetic origin have been studied preclinically. The *WNT* signaling components, mainly *FRZDs* and *DVL*, have been considered as targets for cancer treatment using small-molecule inhibitors. OMP-18R5 (vantictumab) is an antibody that interferes with the binding of *WNT* ligands to *FRZDs*. It has shown activity to inhibit the growth of a variety of human tumor xenograft models and exhibits activity when treated with standard ChT (Table 3) [82].

Another target of *WNT* signaling is porcupine, an enzyme necessary for palmitoylation of *WNT* ligands. Porcupine inhibitory molecules IWP2 and C59 have shown potent in vitro and in vivo activity, respectively. Furthermore, LGK979, another porcupine inhibitor, has demonstrated activity inhibiting *WNT* signaling *in vivo* at well-tolerated doses and laid the foundation for the treatment of *WNT*-driven tumors in the clinic [163]. Currently, a phase I study of LGK979 (NCT01351103) is being conducted to find LGK979 safe doses for melanoma and other solid malignancies (last update posted, 27 July 2021; clinicaltrials.gov, accessed date, 6 August 2021).

On the other hand, potential therapeutic strategy targeting *WNT* signaling includes the development of specific peptides that mimic WNT proteins, such as *WNT3A* and *WNT5A*, and, thus, lead to receptor inhibition. So far, it has shown efficacy in melanoma preclinical models and used in other diseases with promising results [164]. Ultimately, target-specific therapies have also been proposed against extra- and intracellular components of the *WNT* pathway, including axin, *APC*, and *β-catenin* [165]. Along the same line, antibody-based blockage of *WNT5* has resulted in the inhibition of protein kinase C activity and a decrease in cell invasion [166].

## 5. Conclusions and Future Directions

Targeting the *MAPK* signaling pathway has significantly improved the treatment of metastatic melanoma, with *BRAF* mutations being the most frequent and most important alterations to be treated. Targeted therapy for patients whose tumors harbor the *BRAF*V600 mutation achieves high response rates and OS benefit with the combination *BRAF/MEK* inhibition and represents the ideal first-line treatment for patients with *BRAF*-mutated advanced melanoma. However, despite good treatment responses, drug resistance is very common.

To avoid resistance, combination treatments have been and are continuously being studied. The highly complex interactions between melanoma molecular pathways include poorly understood mechanisms that promote drug resistance and decrease patient survival, primarily by activating the *MAPK* and *PI3K-AKT* pathways. Preclinical studies looking at these major drug association strategies appear to be, at least, very promising as a target for *MEK* or *PI3K/mTOR*, blocking *CDK4/6* or RhoA GTPases, which the latter has been shown to promote resensitization to *BRAF*-targeted therapy [96,111]. In the field of other gene alterations, there is a challenge in the research of new therapeutic targets and development of new drugs, especially after failure of immunotherapy with anti-CTLA-4 or anti-PD1 antibodies. Alterations under study that can eventually lead to a therapeutic benefit with targeted therapy are, among others: *NRAS*, *C-KIT*, *GNAQ*, *GNA11*, and *SF3B1*. Moreover, advances have been made in cancer therapy through the use of ICIs. We now have several different approved regimens, both for targeted therapy for *BRAF*-mutated melanoma, as well as immunotherapy for all comers with melanoma.

Using T cells to control disease, adoptive cell therapy, has added a powerful and novel therapeutic option for the treatment of melanoma (TILS, TCR-T, and CAR-T). Among the strategies that have arisen, it has been shown that with TCR-T, the selection of a single antigen it is unlikely to be sufficient to eliminate solid tumors. Therefore, future studies targeting multiple antigens simultaneously may improve the efficacy of TCR-T cells in solid tumors, as has been demonstrated in preclinical models. On the other hand, CAR-T cell therapy could be used for patients with melanoma who are resistant to other therapeutic choices.

In this review, we have emphasized the understanding of the molecular pathways responsible for the development and progression of melanoma, as well as the relevance of detecting the specific molecular markers of each melanoma subtype. This information is of primary importance in clinical practice to predict the response to treatment in each subtype of melanoma.

The different molecular pathways for the development and progression of melanoma are very complex and interact with each other (via crosstalk mechanisms) to create resistance to treatment and the progression of cell signaling. For this reason, there is an urgent need to identify other alternative and targeted therapies. Detailed understanding of the role of genes and proteins in key signaling pathways in melanoma development has led to the designation of new targets for the treatment of melanoma. Recently, analysis of CRISPR-CAS9 screens has identified genes that have not previously been associated with melanoma growth and that can be targeted using available inhibitors, thus opening new treatment strategies that may be explored in the near future as potential therapeutic targets [167]. Furthermore, in the future, more clinical trials and more data on OS and response rates need to be collected to find the best combination treatment and the best possible sequence of combination therapy to manage the complexity of melanoma treatment.

## Figures and Tables

**Table 1:** The classification of melanomas (modified from 2018 World Health Organization Classification).

UV Exposure	Categories	Melanoma Subtype	Key Molecular Genes	
Low UV/CSD	I	Superficial spreading melanoma	*BRAF*V600 mut *CDKN2A* mut *NRAS* mut	*TERT* mut *PTEN* mut *TP53* mut
High UV/CSD	II	Lentigo maligna melanoma	*NRAS* mut *BRAFnon-V600E* mut *KIT* mut *TERT* mut	*CDKN2A* mut *PTEN* mut *TP53* mut
	III	Desmoplastic melanoma	*NF1* mut *NFKBIE* mut	*NRAS* mut *PIK3CA* mut
Low or no UV/CSD	IV	Spitz melanoma	*ALK* rearr *NTRK1* rearr *NRTK3* rearr	*CDKN2A* mut *HRAS* mut
	V	Acral melanoma	*KIT* mut *NRAS or BRAF* mut *ALK* rearr *NRTK3* rearr	*CDKN2A* mut *CCND1* amp *TERT* mut
	VI	Mucosal melanoma	*KIT* mut *NRAS or BRAF* mut *CDKN2A* mut *SF3B1* mut	*CCND1* amp *CDK4* mut *MDM2* amp
	VII	Melanoma in congenital nevus	*NRAS* mut	*BRAFV600E* mut
	VIII	Melanoma in blue nevus	*GNA11* mut *GNAQ* mut *CYSLTR2* mut	*BAP1* mut *EIFAX* mut *SF3B1* mut
	IX	Uveal melanoma	*GNA11* mut *GNAQ* mut *CYSLTR2* mut *PLCB4* mut	*BAP1* mut *EIFAX* mut *SF3B1* mut

Abbreviations: amp, amplification; CSD, cumulative sun damage; mut, mutation; rearr, rearrangement.

**Table 2:** Targeted therapy studies in melanoma patients with impact on clinical practice.

Trial Name	Phase	Patients	Treatment Groups	Primary Endpoint	*n*	ORR	PFS	OS	Reference
		**Anti-CTLA-4**							
MDX010-020 (NCT00094653)	III	Untreaded MM	Ipi 3 + gp100 vs. Ipi 3 vs. gp100 (3:1:1)	OS	676	6 vs. 11 vs. 2	2.8 vs. 2.9 vs. 2.8	10.0 vs. 10.1 vs. 6.4	[48]
		**Anti-PD1 +/− anti-CTLA-4**							
CM-066 (NCT01721772)	III	Untreated *BRAF*- wild type MM	Niv 3 q2w + Placebo vs. Placebo + Dacarbazine 1000 q3w (1:1)	OS	418	40 vs. 14	5.1 vs. 2.2	37.5 vs. 11.2	[49]
CM-067 (NCT01844505)	III	Untreated MM	Niv 1 + Ipi 3 (q3w) x4 − Niv 3; Niv 3 alone q2w, vs. Ipi 3 q3w x4 (1:1:1)	PFS and OS co-primary	945	58 vs. 44 vs. 19	11.5 vs. 6.9 vs. 2.9		[50]
CM-511 (NCT02714218)	III	Untreated MM	Niv 1 + Ipi 3 (q3w) x4 − Niv 3 vs. Niv 3 +Ipi 1 (q3w) x4 − Niv 3 (1:1)	TRAE rate (grade 3–5)	360	48 vs. 34	8.9 vs. 9.9	NR vs. NR	[51]
KN-006 (NCT1866319)	III	MM ≤ 1 line (anti-PD1/PD-L1+/− anti-CTLA-4 included)	Pem 10 q2w vs. Pem 10 q3w vs. Ipi 3 q3w (1:1:1)	PFS and OS (co-primary)	834	34 vs. 33 vs. 12	8.4 vs. 3.4	32.7 vs. 15.9	[52]
		**BRAFi monotherapy**							
BRIM-3 (NCT01006980)	III	Untreated MM	Vem 960 mg bd vs. DTIC (1:1)	PFS and OS (co-primary)	675	48 vs. 5	5.3 vs. 1.6	13.6 vs. 9.7	[9]
BREAK3 (NCT01227889)	III	Untreated *BRAF*V600E MM	Dab 150bd vs. DTIC (3:1)	ORR	250	50 vs. 6	6.9 vs. 2.7	20 vs. 15.6	[53]
		**Combined BRAFi + MEKi**							
COMBI-v (NCT01597908)	III	Untreated *BRAF* V600E/K MM	Dab 150 bd + Tra 2 od vs. Vem 960 bd (1:1)	OS	704	64 vs. 51	11.4 vs. 7.3	NR vs. 17.2	[54]
COMBI-d (NCT01584648)	III	Untreated *BRAF* V600E/K MM	Dab 150 bd + Tra 2 od vs. Dab 150 bd (1:1)	PFS	423	69 vs. 53	11.0 vs. 8.8	25.1 vs. 18.7	[55]
CoBRIM (NCT01689519)	III	Untreated *BRAF*V600 MM	Cob 60 od d1-21 + Vem 960 bd vs. Vem 960 bd + Placebo (1:1)	PFS	495	68 vs. 45	12.3 vs.7.2	22.3 vs. 17.4	[56]
COLUMBUS (NCT01909453)	III	Untreated *BRAF* V600E/K MM	Enc 450 od + Bin 45 mg bd vs. Enc 300 mg od vs. Vem 960 mg bd (1:1:1)	PFS	577	64 vs.52 vs. 41	14.9 vs. 9.6 vs. 6.3	33.6 vs. 23.5 vs. 16.9	[57]
		**Triplet therapy (ICI + BRAFi + MEKi)**							
IMSpire150 (NCT02908672)	III	Untreated *BRAF*V600 MM	Ate 840 d1,15 + Vem 720 bd + Cob 60 od d1-21 vs. Placebo + Vem 960 bd + Cob 60 od d1-21 (all: q4w)	PFS	514	66 vs. 65	15.1 vs. 10	Not yet reported	[58]
COMBI-I (NCT02967692)	III	Untreated *BRAF*V600 MM	Spa 400 mg + Dab 150 bd + Tra 2 od vs. Placebo + Dab 150 + Tra 2 (q4w)	PFS	532	69 vs. 64	16.2 vs. 12.0	NR vs. NR	[59]

Abbreviations: Ate, atezolizumab; bd, twice daily; Bin, binimetinib; Cob, cobimetinib; Dab, dabrafenib; Enc, encorafenib; Ipi, ipilimumab; IT, immunotherapy; MM, metastatic melanoma; Niv, nivolumab; NR, not reached; nr, not reported; od, once daily; ORR, overall response rate; OS, median overall survival; Pem, pembrolizumab; PFS, median progression-free survival; q2w/q3w/q4w, all two/three/four weeks; Spa; spartalizumab; Tra, trametinib; TRAE, treatment-related adverse events; Vem, vemurafenib; vs. versus.

**Table 3:** Potential targets in melanoma.

The Key Type of Target Mechanism	Potential Agen/Drugs	Phase	Potential Clinical Indication	ORR (%)	References
*VEGFR1*–*VEGFR3*, *C-KIT*, *PDGFR*	Axitinib	II Ib	Monotherapy in advanced melanoma In mucosal melanoma in combination with toripalimab (anti-PD1)	18.8 48.3	[60] [61]
*VEGFR1*–*VEGFR3*; *FGFR1-FGFR3*; *PDGFR*; *C-KIT*; and *RET*	Lenvatinib	I Ib/II	Monotherapy in advanced melanoma In advance melanoma in combination with pembrolizumab	17.2 48	[62] [63]
*C-KIT* inhibitor	Imatinib, Nilotinib, Dasatinib	II	Studied in mucosal, acral, and chronically sun-damaged melanomas	23.3–26.2	[64,65,66]
*IGF*-1 inhibitor	Linsitinib	I	In combination with erlotinib in solid tumors	1/1	[67]
*EGF* inhibitor	Gefitinib, Erlotinib	II, I	Minimal clinical efficacy as a single-agent therapy for unselected patients with metastatic melanoma. In combination with pictilisib (*PI3K* inhibitor) in solid tumors	3.5–4	[68,69]
*VEGF* inhibitor	Bevacizumab	II	In combination with dacarbazine for the treatment of unresectable/metastatic melanoma In combination with temozolomide as the first line of treatment metastatic uveal melanoma	18.9 0	[70] [71]
*MEK* inhibitor	Pimasertib Selumetinib	II I	Monotherapy in NRAS-mutated melanoma Monotherapy in comparison to temozolamide in chemo-naive stage unresectable III/Vmelanoma	23 5.8	[72,73,74]
*PI3K/mTOR* dual inhibitor	Voxtalisib	Ib	Tested in combination with pimasertib in melanoma patients with genetic alteration in *PTEN*, *BRAF*, *NRAS*, *KRAS*, *PI3KCA*, *ERBB1/2*, *RET*, *MET*, *KIT*, *GNAQ*, *GNA11*, but with limited antitumor activity and tolerance	6	[75]
*PI3K* inhibitor	Pictilisib	I	In combination with erlotinib in solid tumors or alone	3.5–22	[68,76]
*mTOR* inhibitor	Everolimus	I	In combination with *VEGFR* kinase inhibitor (vatalanib) for patients with advanced solid tumors	12.9	[77]
	Temsirolimus	II	Tested in combination with sorafenib Clinical activity of combination therapy with temsirolimus plus bevacizumab, which may be greater in patients with *BRAF* wild-type melanoma	5 17.7	[78,79]
*AKT* inhibitor	Uprosertib (GSK2141795)	I	In combination with trametinib in patients with advanced *BRAF* wild-type melanoma and triple-negative breast cancer	<5	[80]
*AKT* inhibitor	Afuresertib	I	In combination with the *MEK* inhibitor trametinib in patients with solid tumors and multiple myeloma	5	[81]
Wnt inhibitor	Vantictumab (OMP-18R5)	NA	Have shown antitumor growth in xenograft models, particularly in combination with standard chemotherapeutic agents, have not reached clinical trial	NA	[82]
	LGK974	I	Monotherapy or in combination with PDR001 in patients with solid tumors (recruiting)	NA	NA
*IKK* inhibitor	BMS-345541	NA	A proposed target drug but have not reached clinical trial	NA	[83]
*MITF* promoter: HDAC inhibitors	Panobinostat	I	Tested in patients with metastatic melanoma	0	[84]
*CDK4/6* inhibitor	Palbociclib	II I/II	Monotherapy in patients with advanced acral lentiginous melanoma with CDK pathway gene aberrations (*CDK4* or/and *CCND1* amplification or/and *CDKN2A* loss) In combination with vemurafenib in *BRAF*V600-mutated advanced melanoma patients harboring *CDKN2A* loss and RB1 expression	20 27.8	[85]
*CDK4/6* inhibitor	Abemaciclib	NA	Effective in *BRAF*-resistant melanoma cells, preclinical data	NA	[86]
*NTRK* inhibitors	Selitrectinib (BAY 2731954, LOXO-195); Repotrectinib	NA	The second generation of *NTRK* designed to address on-target resistance, preclinical data on *ROS1*-, *NTRK1-3*-, or *ALK*-rearranged malignancies	NA	[87,88]
*ALK* inhibitors	Ceritinib Crizotinib	NA I	In vivo and in vitro studies showed that mucosal melanomas expressing *EML4-ALK* fusions are sensitive to *ALK* inhibitors Crizotinib in combination with vemurafenib in advanced *BRAF*-mutated tumors, mostly melanoma	11 29	[89] [90]
*SF3B1 inhibitors*	E7107	I	Monotherapy in solid tumors	0	[91,92]

Abbreviation: NA, not available; ORR, overall response rate.

## Data Availability

Not applicable.

## Abbreviations

ACT	adoptive cell therapy;
AE	adverse events;
*ALK*	Anaplastic lymphoma kinase;
*BAD*	*BCL-2* antagonist of cell death
BRAF	v-raf murine sarcoma viral oncogene homolog B1;
*BRAF*	inhibitor (*BRAF*i)
CAR-T	chimeric antigen receptor T cells;
*CCND1*	cyclin D1;
*CDK4*	cyclin-dependent kinase 4;
CDKN2A	cyclin-dependent kinase inhibitor 2 receptor;
ChT	chemotherapy;
CSD	cumulative sun damage;
CTLA-4	cytotoxic T-lymphocyte–associated antigen 4;
*CTNNB1*	Catenin Beta 1;
cuSCC	cutaneous squamous cell carcinoma;
DVL	dishevelled;
*ERK*	extracellular signal-related kinase;
*FAMMM*	familial atypical multiple mole-melanoma;
FDA	US Food and Drug Administration;
*FRZD*	Frizzled receptors;
GEP	gene expression profiles;
GPCR	G-protein-coupled receptors;
*GSK3β*	glycogen synthase kinase 3β;
GTPase	guanosine triphosphatases;
*HRAS*	v-Ha-ras Harvey rat sarcoma viral oncogene homolog;
ICI	immune checkpoint inhibitor;
*IFNG*	interferon-gamma;
IL	interleukin;
iNOS	inducible nitric oxide synthase;
*JNK*	c-Jun N-terminal kinases;
*KRAS*	v-Ki-ras2 Kirsten rat sarcoma viral oncogene homolog;
*LEF*	lymphoid enhanced transcription factor;
*LRP*	lipoprotein receptor;
MAPK	mitogen-activated protein kinase;
*MAP2K1*	mitogen-activated protein kinase kinase 1;
*MAP3K1*	mitogen-activated protein kinase kinase kinase 1;
*MC1R*	melanocortin 1 receptor;
MDM2	Mouse double minute 2 homolog;
*MEK*	inhibitors (*MEK*i)
*MITF*	microphthalmia-associated transcription factor;
*mTOR*	mammalian target of rapamycin;
*NBD*	NEMO-binding domain;
*NF1*	neurofibromin 1;
*NFκB*	nuclear factor-kappaB;
*NTRK*	Neurotrophic Tyrosine Receptor Kinase;
*NRAS*	neuroblastoma ras viral oncogene homolog;
ORR	overall response rate;
OS	overall survival;
PD1	programmed cell death-1;
PD-L1	programmed cell death ligand-1;
*PI3K*	phosphatidylinositol-3-kinase;
PIP2	phosphatidylinositol-4,5-diphosphate
PIP3	phosphatidylinositol-3,4,5-trisphosphate
*PTEN*	phosphatase and tensin homolog;
RTK	receptor tyrosine kinase;
*SF3B1*	Splicing Factor 3b Subunit 1;
TCR-T	T-cell receptor– engineered T cells;
*TERT*	Telomerase Reverse Transcriptase;
TILS	Tumor-infiltrating lymphocytes;
TCF	T-cell transcription factor;
TCGA	The Cancer Genome Atlas
TNF	tumor necrosis factor;
TRAE	treatment-related adverse event
*TRK*	tropomyosin receptor kinases;
UV	ultraviolet;
WHO	World Health Organization
